# Genetic parameters and correlations of related feed efficiency, growth, and carcass traits in Hanwoo beef cattle

**DOI:** 10.5713/ajas.20.0135

**Published:** 2020-08-30

**Authors:** Hossein Mehrban, Masoumeh Naserkheil, Deuk Hwan Lee, Noelia Ibáñez-Escriche

**Affiliations:** 1 Animal Science, Shahrekord University, Shahrekord, Charmahalo-Bakhteiari 88186-34141, Iran; 2 Animal Science, University of Tehran, Tehran 77871-31587, Iran; 3 Animal Life and Environment Sciences, Hankyong National University, Anseong 17579, Korea; 4 Institute for Animal Science and Technology, Universitat Politècnica de València, València 46022, Spain

**Keywords:** Feed Efficiency, Growth Traits, Carcass Traits, Genetic Correlation, Hanwoo Cattle

## Abstract

**Objective:**

This study aimed to estimate the genetic parameters and genetic correlations for related feed efficiency, growth, and carcass traits in Hanwoo cattle.

**Methods:**

Phenotypic data from 15,279 animals born between 1989 and 2015 were considered. The related feed efficiency traits considered were Kleiber ratio (KR) and relative growth rate (RGR). Carcass traits analyzed were backfat thickness (BT), carcass weight, eye muscle area, and marbling score. Growth traits were assessed by the average daily gain (ADG), metabolic body weight (MBW) at mid-test age from 6 to 24 months, and yearling weight (YW). Variance and covariance components were estimated using restricted maximum likelihood using nine multi-trait animal models.

**Results:**

The heritability estimates for related feed efficiency (0.28±0.04 for KR and RGR) and growth traits (0.26±0.02 to 0.33±0.04) were moderate, but the carcass traits tended to be higher (0.38±0.04 to 0.61±0.06). The related feed efficiency traits were positively genetically correlated with all the carcass traits (0.37±0.09 to 0.47±0.07 for KR, and 0.14±0.09 to 0.37±0.09 for RGR), except for BT, which showed null to weak correlation. Conversely, the genetic correlations of RGR with MBW (−0.36±0.08) and YW (−0.30±0.08) were negative, and those of KR with MBW and YW were close to zero, whereas the genetic correlations of ADG with RGR (0.40±0.08) and KR (0.70±0.05) were positive and relatively moderate to high. The genetic (0.92±0.02) correlations between KR and RGR were very high.

**Conclusion:**

Sufficient genetic variability and heritability were observed for traits of interest. Moreover, the inclusion of KR and/or RGR in Hanwoo cattle breeding programs could improve the feed efficiency without producing any unfavorable effects on the carcass traits.

## INTRODUCTION

The global interest in improving the profitability of beef cattle production systems has recently increased. It is therefore important to identify animals that can efficiently utilize feed resources for reducing feeding costs and improving feed efficiency. Presently, several economically important traits, including body weight, weight gains at specific ages or during specific periods, carcass traits, feed intake, and feed efficiency, are commonly considered as selection criteria in most beef cattle breeding programs [1–3].

Measures of feed efficiency, including residual feed intake [4], feed conversion ratio (FCR) [5], relative growth rate (RGR) [6], and Kleiber ratio (KR) [7] provide possible means for improving the profitability of beef cattle production systems. Selection for KR and RGR can be considered as indirect feed utilization measures that can improve the feed efficiency traits with no individual record on feed intake [6,8]. Studies have reported strong negative genetic correlations of the FCR with RGR (−0.56 and −0.90) and KR (−0.74 and −0.81) in Japanese Black cattle [1] and young Charolais bulls [9], respectively. Studies have also reported the estimates for some genetic parameters for feed efficiency traits in Irish cattle [10] and their correlations with growth traits in Nellore cattle [3]. In contrast, although several studies have investigated the genetic parameters estimates for carcass traits in Hanwoo cattle [11–13], no estimates of genetic parameters for feed efficiency traits are currently known for this breed.

Feed efficiency has been especially emphasized in Korea since high proportions of feeding resources depend on imports owing to the shortage of roughage and grain production. Nevertheless, the Hanwoo beef industry primarily aims to increase the quantity (carcass weight [CW]) and quality (backfat thickness [BT]; eye muscle area [EMA]; and marbling score [MS]) of the meat, without considering the inclusion of feed efficiency traits in the selection indices for selecting young bulls or/and proven bulls [14]. It is therefore important to understand the genetic basis of the traits related to feed efficiency (*e.g*. KR and RGR) and to compute the magnitude of the correlations of these traits with carcass and growth traits, which will allow the construction of an optimal multiple-trait selection index for increasing the profitability of beef production.

To accomplish these goals, we evaluated the genetic parameters for measures of related feed efficiency, including RGR and KR, for the first time, as well as measuring the phenotypic and genetic correlations between i) feed efficiency and growth traits, and ii) feed efficiency and carcass traits in Hanwoo beef cattle.

## MATERIALS AND METHODS

### Phenotypic and pedigree data

Records of 15,279 animals (8,966 bulls and 6,313 steers) born between 1989 and 2015 from the Hanwoo Improvement Center of the National Agricultural Cooperative Federation were used for this study (Table 1). The pedigree data of 50,127 animals, obtained after tracing the pedigree file back to 11 generations, were employed in the animal model. MS was measured using a categorical system of nine classes ranging from the lowest score of one (no marbling) to the highest score of nine (abundant marbling). The records on MS collected before 2005 were removed owing to mismatches with the newly adopted 9-point MS scoring system. The carcass traits were measured according to the Korean carcass grading system in steers at approximately 24 months of age, ribbed between the 13th rib and the first lumbar vertebrae, 24 h postmortem, according to notification No. 2014-4 of the Ministry of Agriculture, Food and Rural Affairs.

Yearling weight (YW) for each animal was determined from the weight (W_t_) at the termination (t) of the test (body weight at ~12 months) and the previous weight (W_t−1_) recorded at a time point (t_−1_) before (body weight at ~6 months) the termination, according to the equation described by Park et al [14]:

$$\text{YW}=(\frac{{\text{W}}_{\text{t}}-{\text{W}}_{\text{t}-1}}{\text{t}-t_{-1}})\times (365-t_{-1})+{\text{W}}_{\text{t}-1}$$

A linear regression of body weights recorded at around 6, 12, 18, and 24 months was used to estimate the average daily gain (ADG), according to the following equation:

$${\text{Y}}_{1}=\beta_{0}+\beta_{1}{\text{x}}_{1}+{ɛ}_{1}$$

Where y represents the weight of the animal, β_0_ is intercept, β_1_ is the linear regression coefficient that represents ADG, x is the age in days, and ɛ is the random residual effect.

The mid-test age (x*) and the mid-test metabolic body weight (MBW) were obtained by the following equation: $\left({\text{age}}_{\text{min}}+\frac{\left[{\text{age}}_{\text{max}}-{\text{age}}_{\min}\right]}{2}\right)$and (β_0_ + β_1_x^*^)^0.75^, respectively; where age_min_ and age_max_ are the minima and maximum recorded ages for each animal. RGR was calculated using the following equation:

$$\text{RGR}=\frac{\left(\text{log}\;\text{FBW}-\text{log}\;\text{IBW}\right)}{\left({\text{age}}_{\text{max}}-{\text{age}}_{\min}\right)}\times 100$$

in which FBW and IBW represent the final and initial body weights in addition to different maximum and minimum ages is based on year. KR was obtained as a ratio of ADG to mid-test MBW.

### Statistical analyses

The national genetic evaluation of Hanwoo considers YW as one of the traits for selecting young bulls at the performance test stage. Therefore, the YW of all the candidate young bulls are recorded, but the body weight traits at 15, 18, and 24 months, and the carcass traits are measured only for the offspring of the young bulls that are selected for producing steers [14]. Hence, analyses of the carcass traits, ADG, MBW, KR, and RGR will be biased unless the data for YW is included. A multi-trait analysis that includes YW and these traits can remove this bias [15]. Hence, the nine multi-trait analysis was performed in REMLF90 [16], is given as follows:

y=Xb+Zu+e

where **y** is the vector representing the observations for the traits of interest; **b** is the vector representing the fixed effects, including batch-test place-sex (163 levels for YW and 48 levels for ADG, MBW, KR, and RGR) and birth place (108 levels for YW and 85 levels for ADG, MBW, KR, and RGR); batch-test place-slaughter date (391 levels; only for MS, 201 levels), birth place (86 levels; only for MS, 76 levels), and slaughter age (days from birth to slaughter) as covariates for carcass traits (except for BT, owing to the lack of significant effects of slaughter age); **u** is the vector of random genetic additive effects; **e** is the vector of random residual effects; and **X** and **Z** are incidence matrices related to the fixed and random genetic additive effects, respectively. Var (**u**) = **G⊗A** and Var(e) = **R⊗I**, were assumed, where **A** is the numerator relationship matrix, **I** is the identity matrix, and **G** and **R** are additive genetic and residual covariance, respectively, for the two or three traits.

Indeed, a multi-trait analysis can be problematic as the number of traits analyzed increase particularly in the presence of wrong starting values for variance-covariance parameters. Hence, the reasonable starting values for genetic and environmental variance-covariance among the nine traits were considered using a series of two-trait analyses according to above the equation. Finally, the pooled estimates of (co)variances were obtained with the option “--pool” in Wombat software [17] because of different datasets; then they were defined as starting values in the parameter file to estimate (co) variance components using nine multi-trait animal model.

REMLF90 [16] software cannot estimate the standard errors (SEs) for the (co)variance components and heritabilities; therefore, they were estimated by repetitive sampling, performed 10,000 times, of the nine-trait estimates of (co)variances in AIREMLF90 [16] software.

In addition, a single-trait analysis was performed using for each trait to obtain initial values of variances and covariances for subsequent restricted maximum likelihood multi-trait analyses.

## RESULTS AND DISCUSSION

Recently, the characterization of economically important traits and their associations has been of great interest in developing effective genetic improvement programs to increase the overall profitability of beef cattle production systems. In the present study we evaluated, for the first time, the genetic parameters of KR and RGR (indirect measures of feed efficiency) and their correlations with growth and carcass traits in Hanwoo beef cattle.

### Variance components and heritability estimates

Table 2 shows estimates of the variance components and heritability for the studied traits. The heritability estimate (±SE) for MS was the highest (0.61±0.06), whereas those for CW, EMA, and BT were moderate to high, being 0.38± 0.04, 0.43±0.04, and 0.50±0.05, respectively. The estimated heritabilities for the growth traits, ADG, MBW, and YW, were 0.33±0.04, 0.33±0.03, and 0.26±0.02, respectively. RGR and KR had moderate heritability of 0.28±0.04. Our results showed considerable additive genetic variation (based on CV_g_) for MS and BT.

In the present study, the heritability estimates for carcass traits were in accordance with most of the previous studies in Hanwoo cattle [12,14,18], except those of Kim et al [11] and Bhuiyan et al [13]. These disagreement in the heritability estimates may be due to differences in slaughter age of animals measured which likely leading to differences in the structure of data among Hanwoo populations. In comparison with other breeds, our estimate of heritability for CW was lower than those reported in other breeds as Brahman [19,20] and Japanese Black cattle [21]. Similarly, the heritability estimated for BT was lower than the results reported by Riley et al [19] for Brahman (0.63), and Takeda et al [21] for Japanese Black cattle (0.57) but similar to that reported by Yokoo et al [22] for Nellore animals (0.50). For EMA, other studies reported lower estimates (0.29) [22,23] for Nellore or higher heritability (0.59) measures for Japanese Black [21], in comparison to the results of our study. Regarding MS, although our estimation of the heritability (0.61) was high, it was lower than that obtained by Takeda et al [21] who estimated the heritability of 0.77 in Japanese Black cattle. Nevertheless, Smith et al [20] and Riley et al [19] showed that the estimated heritability for MS was 0.37 and 0.44, respectively, in Brahman cattle.

Analysis of the growth traits revealed that the heritability for YW (0.26) was slightly lower than that (0.30) obtained by Park et al [14], but higher than the estimate of 0.18 reported by Choi et al [12] in Hanwoo beef cattle. Beside different in statistical models and more animal in pedigree in our study, the number of records for YW increased around 94% and 72% compared with Park et al [14] and Choi et al [12], respectively. Other earlier studies by Zuin et al [23] and Yokoo et al [22] reported heritability estimates of 0.29 and 0.32, respectively, for YW in Nellore cattle, while Kemp et al [24] obtained a heritability of 0.55 for Angus cattle.

The heritability estimated for ADG (0.33) was in line with the literatures [10,25–28]; but lower than some previously reported estimates of 0.44 [3] and 0.54 [21]. Our heritability estimate for MBW (0.33) was similar to the estimate of 0.35 reported by Schenkel et al [26], but lower than the reported in other studies by Arthur et al [25], Crowley et al [10], Grion et al [3], and Ceacero et al [29], which were 0.40, 0.43, 0.53, and 0.53, respectively.

Heritability estimates for the related feed efficiency traits, KR and RGR (0.28), were slightly lower than that those reported by Arthur et al [9] in Charolais cattle of 15 months of age (0.30 and 0.31) and Coyne et al [30] in Irish cattle (0.31 and 0.33). Nevertheless, our present estimates were somewhat higher than the estimates obtained in earlier studies [1,3,10,31], which ranged from 0.14 to 0.24. The heritability estimated jointly with genetic CV for KR (4.57%) and RGR (6.13%) would indicate that these traits would be effective for genetic improvement.

Additionally, the results showed that the heritabilities using single-trait were lower than those obtained by multi-traits for BT, CW, ADG, and MBW; while, similar heritabilities were observed for EMA, MS, and YW (Supplementary Table S1). However, heritabilities of KR and RGR were slightly decreased when the model changed from single to multi-trait. The reason for the discrepancy between the results from single-trait and multi-trait analyses was probably due to exploiting the genetic correlation among the traits in the multi-trait compared to the single-trait model. It is interesting to note that the use of multivariate models is known to provide more accurate breeding values, better connections in the data due to residual covariance between traits and avoiding culling bias than those obtained by single-trait models, as the information from genetically correlating traits can be utilized [15].

In general, our heritability results were in the range of the heritabilities previous reported in Hanwoo cattle and in most of the beef cattle studied. Nevertheless, the differences founded could be attributed to the differences in the number of animals considered, breed, the completeness of the pedigree, precision of recording, environmental variation, and statistical models used for analyses.

### Correlations among the traits

Table 3 shows the results of the genetic and phenotypic correlations among the carcass traits. The EMA showed a moderate, positive genetic correlation with CW (0.55±0.06), whereas the genetic correlations of EMA with the two other carcass traits, BT and MS, were −0.11±0.08 and 0.28±0.08, respectively. Positive, but very low additive genetic correlations, were observed between CW and MS (0.16±0.08), and between CW and BT (0.18±0.07). However, the additive genetic correlation between BT and MS (−0.03±0.08) was nearly zero. The estimated phenotypic correlations between CW and two other carcass traits, EMA and BT, were significantly positive, being 0.57±0.01 and 0.30±0.01, respectively, unlike the correlation between CW and MS, which was very low (0.11±0.02). Additionally, we observed a positive phenotypic correlation between EMA and MS (0.21±0.02), whereas the correlation of BT with both MS (0.07±0.02) and EMA (0.03±0.02) was negligible.

Table 4 summarizes the genetic and phenotypic correlations of the carcass traits with the growth and related feed efficiency traits. Compared to the other carcass traits, strong additive genetic correlations were observed between CW and the three growth traits (ADG, MBW, and YW), ranging from 0.74±0.04 to 0.94±0.01, with ADG showing the highest correlation. BT and MS showed weak genetic correlations with all the growth traits, ranging from 0.00±0.08 to 0.11± 0.08 for BT and from −0.16±0.08 to 0.19±0.08 for MS, whereas the genetic correlations of EMA with YW (0.33±0.07), ADG (0.58±0.06), and MBW (0.40±0.07) were low to moderate. Phenotypically, CW was positively and strongly correlated (0.70±0.01 to 0.84±0.00) with all the growth traits, however, the phenotypic correlations of these growth traits with MS were close to zero (Table 4). Nonetheless, all the growth traits had very low to relatively moderate, positive phenotypic correlations with BT and EMA and ranged from 0.17±0.01 to 0.47±0.01 (Table 4). The genetic correlations of KR and RGR with the three carcass traits (CW, EMA, and MS) were positive, ranging from 0.14±0.09 to 0.47±0.07 (Table 4). In contrast, BT had a very low, negative genetic correlation with KR (−0.02 ±0.09) and RGR (−0.11±0.09). Additionally, the phenotypic correlations of RGR and KR with all the carcass traits were very low.

Table 5 depicts the genetic and phenotypic correlations among the growth and related feed efficiency traits. Analysis of the relationship among the growth traits revealed that YW had strong, positive genetic (0.89±0.02) and phenotypic correlations (0.85±0.00) with MBW. The magnitude of the association between YW and ADG was positive and high, with genetic and phenotypic correlations of 0.61±0.06 and 0.49±0.01, respectively. Similarly, significant correlations were observed between ADG and MBW at the genetic (0.70 ±0.05) and phenotypic levels (0.63±0.01). Analysis of the association between the feed efficiency traits revealed that the genetic and phenotypic correlations between KR and RGR were positive and strong, being 0.92±0.02 and 0.92±0.00, respectively. The genetic and phenotypic correlations between ADG with RGR (0.40±0.08 and 0.45±0.01) and KR (0.70± 0.05 and 0.71±0.01) were positive and relatively moderate to high. Nevertheless, low genetic and phenotypic correlations of KR with the two other growth traits, MBW and YW, were also estimated. Low, negative genetic associations were observed for the correlations of RGR with MBW (−0.36±0.08) and YW (−0.30±0.08), in addition to low, negative phenotypic correlations (−0.37±0.01 for MBW and −0.30±0.01 for YW) (Table 5).

Analysis of the phenotypic and genetic correlations among the carcass traits agree with the previous study in Hanwoo cattle carry out by Choi et al [12]. However, the weak, negative genetic correlation observed between BT and EMA (−0.11) in the present study are inconsistent with the findings of previous studies in other breeds. For instance, Smith et al [20] reported very low negative genetic correlation (−0.25) for Brahman cattle and Hoque et al [27] reported a highly negative genetic correlation between BT and EMA (−0.99) for Japanese Black steers, whereas Oikawa et al [28] reported a correlation of 0.40. In contrast, the genetic correlation between BT and MS (−0.03) was in the line with most studies in other breeds [20,27] with the exception of the studies by Koots et al [31] and Riley et al [19], wherein positive relationships (0.36 and 0.56, respectively) were observed. The ratio weight of MS to BT in the selection index used for selecting proven bulls is 6:1 [14]; therefore, the weak, negative or almost no genetic correlation between BT and MS observed in the current study represents a favorable association for further improvement of beef marbling, since it means that MS can be achieved without increasing subcutaneous fat, as these traits appear to be genetically independent.

As depicted in Table 3, the moderate, positive genetic correlations (0.55) between CW and EMA obtained in our study are similar to the results of Choi et al [12] and Bhuiyan et al [13], which were 0.52 and 0.60, respectively, but disagree with the findings of Kim et al [11] and Do et al [32], who reported lower (0.07) or greater (0.80) genetic correlations, respectively than our results for Hanwoo cattle. Additionally, positive genetic (0.52 and 0.45, respectively) and phenotypic (0.44 and 0.39, respectively) correlations between CW and EMA shown by others [19,20] in Brahman cattle. The estimated genetic correlations between CW and BT were very low and positive (0.18), which is corroborated by the results of earlier studies (0.17 [24]; 0.16 [11]; 0.17 [32]), but was considerably lower than the estimate of 0.40 reported by Hwang et al [33] in Hanwoo beef cattle. Our results indicated a weak and positive genetic correlation between CW and MS (0.16). However, Kim et al [11] obtained a negative genetic correlation between CW and MS (−0.48) for Hanwoo cattle, which is in contrast to the positive genetic correlations of 0.39 and 0.51 reported by Riley et al [19] and Smith et al [20], respectively, in Brahman cattle. EMA showed positive and relatively low genetic correlation with MS (0.28), which is within the range of the results (0.12 to 0.44) of several previously reported estimates [12,19,20,28]. However, a negative genetic correlation was observed between MS and EMA (−0.40) in an earlier study in Hanwoo cattle [11]. Conversely, Hoque et al [27] obtained a high, positive correlation of 0.72 between MS and EMA in Japanese cattle.

Analysis of the estimated genetic correlations among the growth and carcass traits revealed positive, and low to strong correlations for all the growth traits with both CW and EMA, and especially ADG, which had the greatest correlation with CW (0.94). The results of this study are similar to the earlier observations of Choi et al [12] concerning the genetic correlation between YW and CW, which was reported to have a high magnitude of 0.77, indicating that these traits are under similar genetic control. In our study, the estimated genetic correlation of YW with EMA was low positive (0.33), while the correlations of YW with MS and BT were very low negative (−0.16) and zero, respectively, and are in agreement with the correlations reported by Choi et al [12], who estimated the genetic correlations between YW and the carcass traits, EMA, MS and BT, to be 0.37, −0.19 and −0.03, respectively. However, our estimates of genetic correlation between YW and EMA were lower than those reported by Yokoo et al [22] and Zuin et al [23] for Nellore cattle (0.67 and 0.55, respectively), and that reported by Kemp et al [24] for Angus cattle (0.45). In contrast, the genetic correlation of YW with BT is in the range that reported by Yokoo et al [22] (0.04) and slightly lower than those reported by Kemp et al [24] and Zuin et al [23] (0.10 and 0.15, respectively). The phenotypic correlations obtained between YW and carcass traits in our study are somewhat higher than those described by Choi et al [12]. The genetic correlations of ADG with EMA (0.58) and CW (0.94) were found to be moderate to strongly positive in the present study, consistent with the results of previous studies that reported values of 0.58 and 0.84 for ADG with EMA and CW, respectively [19]. However, other studies reported a correlation of −0.28 [27] and 0.37 [28] between ADG and EMA for Wagyu cattle in Japan. The MS was weak and positively correlated with ADG (0.19), which was lower than the values of the previously reported (0.21 to 0.28) in literature for various breeds of cattle [19,27,31]. The genetic correlation between BT and ADG was negligible, whereas several previous studies have reported positive and higher estimates within a range from 0.49 to 0.54 for BT and ADG [19,27,28]. The phenotypic correlations of ADG with the carcass traits also indicated a similar trend to the genetic correlations and are consistent with findings of earlier studies [19,20,28].

Analysis of the relationship among the related feed efficiency and carcass traits revealed that the genetic correlations of KR and RGR with the three traits, CW, EMA, and MS, were positive but the correlations with BT were weakly negative or close to zero. Hence, selection of animals to improve KR and RGR could lead to increased CW, EMA and MS and reduced BT. Finally, it can be said that the inclusion of KR and/or RGR in the breeding program of Hanwoo cattle could improve feed efficiency without having unfavorable effects on the carcass traits. Hoque et al [1] reported positive genetic correlations for KR and RGR with most of the carcass traits within a range from 0.12 to 0.87, with the exception of the correlations of KR with CW and BT (−0.03 and −0.51, respectively) and RGR with BT (−0.35), which were negative and low to moderate. Another study also reported the weak and positive genetic correlation between KR and CW (0.09) in Irish beef cattle [30].

The genetic relationships among the growth traits observed in this study were positive and high, with YW exhibiting a relatively stronger correlation (0.89) with MBW in particular, in comparison to the other traits. Grion et al [3] observed a high, positive genetic correlation between ADG and MBW (0.74) for Nellore cattle. Substantially similar values for genetic correlation have been reported between ADG and MBW in both Angus (0.77) and Charolais (0.68) steer populations [34], which are consistent with the results of the present study (0.70). In other words, the pressure of selection on YW and ADG traits could increase MBW and results in rising maintenance costs in Hanwoo beef cattle. The low to relatively high genetic correlations of ADG with RGR and KR (0.40 and 0.70, respectively) in this study are consistent with the results of Crowley et al [10] and Grion et al [3]. Additionally, the weak to low, negative genetic correlations of MBW with KR and RGR (−0.02 and −0.36, respectively) observed in our study are in the range those reported by Grion et al [3]. Also, YW showed weak and close to zero genetic correlation with KR (−0.05) and negative low correlation with RGR (−0.30). Although, selection of animals based on RGR and KR would improve ADG; however, RGR could decrease MBW and YW despite KR which is genetically independent of MBW and YW. The strong genetic and phenotypic correlations between KR and RGR in the current study agree with the results of previous studies [1,10,35].

The index for selecting Hanwoo young bulls during performance testing was obtained using SI_YB_ = 2EBV_YW_+EBV_MS_, where, EBV_YW_ and EBV_MS_ are the standardized estimated breeding values for YW and MS (parents average), respectively [14]. As mentioned previously, the genetic correlation of MBW with MS and YW were −0.10 and 0.89, respectively, indicating that a high genetic correlation is expected between MBW and SI_YB_. Increasing the MBW will lead to rising the maintenance requirements and feeding costs, which could be managed by including KR and/or RGR in the young bull selection index.

Information on the genetic and phenotypic correlations of feed efficiency with growth and carcass traits in Hanwoo cattle is scarce, and our findings, notably, are the first reports of some of these estimates for this breed, and will, therefore, be useful in designing breeding programs aimed to improve these traits.

## IMPLICATIONS

The moderate to high heritability estimates for carcass, growth, and related feed efficiency traits, in addition to the sufficient additive genetic variability observed for some of these traits, indicates that further improvement is possible for these traits. Additionally, favorable genetic correlations of Kleiber ratio and relative growth rate with metabolic body weight and carcass traits along with positive and the low to relatively high genetic correlation with average daily gain observed in this study, suggest that the inclusion of related feed efficiency traits in selection programs for Hanwoo cattle is feasible.

## Figures and Tables

**Table 1:** Descriptive statistics for carcass traits, growth traits, and related feed efficiency traits in Hanwoo cattle

Traits (unit)	N	Mean (SE)	SD	Min	Max	CV (%)
Carcass traits						
BT (mm)	5,824	8.71 (0.05)	3.71	1	30	42.61
CW (kg)	5,824	343.96 (0.60)	45.61	158	519	13.26
EMA (cm^2^)	5,821	78.90 (0.12)	9.12	40	123	11.56
MS (1–9)	3,991	3.33 (0.03)	1.61	1	9	48.46
Growth traits						
ADG (g)	6,087	847.34 (1.51)	118.17	387.73	1344.48	13.95
MBW (kg)	6,087	86.68 (0.10)	9.34	53.79	115.08	9.34
YW (kg)	15,279	342.06 (0.38)	47.48	133.86	535.90	13.88
Feed efficiency traits						
KR (g/kg)	6,087	9.78 (0.01)	1.06	5.94	15.23	10.85
RGR	6,087	39.63 (0.07)	5.49	21.00	70.69	13.86

SE, standard error; SD, standard deviation; CV, coefficient of variation; BT, backfat thickness; CW, carcass weight; EMA, eye muscle area; MS, marbling score; ADG, average daily gain; MBW, mid-test metabolic body weight; YW, yearling weight; KR, Kleiber ratio; RGR, relative growth rate.

**Table 2:** Additive genetic variances (σ^2^_a_), environmental variances (σ^2^_e_), phenotypic variances (σ^2^_p_), heritability (h^2^), and coefficients of genetic variation (CV_g_) estimates (±SEs) for carcass, growth, and related feed efficiency traits in Hanwoo cattle

Trait1	σ^2^_a_	σ^2^_e_	σ^2^_p_	h^2^	CV_g_ (%)
BT	5.60 (0.59)	5.70 (0.47)	11.29 (0.25)	0.50 (0.05)	27.17
CW	470.90 (48.90)	782.50 (40.29)	1,253.40 (24.70)	0.38 (0.04)	6.31
EMA	28.36 (3.17)	37.51 (2.59)	65.87 (1.44)	0.43 (0.04)	6.75
MS	1.54 (0.18)	0.98 (0.14)	2.52 (0.07)	0.61 (0.06)	37.27
ADG	2,759.00 (321.92)	5,523.00 (273.12)	8,282.00 (162.28)	0.33 (0.04)	6.20
MBW	13.92 (1.39)	28.01 (1.14)	41.93 (0.71)	0.33 (0.03)	4.30
YW	265.70 (25.02)	765.90 (20.26)	1,031.60 (13.56)	0.26 (0.02)	4.77
KR	0.20 (0.03)	0.50 (0.02)	0.69 (0.01)	0.28 (0.04)	4.57
RGR	5.91 (0.82)	15.32 (0.72)	21.23 (0.41)	0.28 (0.04)	6.13

BT, backfat thickness; CW, carcass weight; EMA, eye muscle area; MS, marbling score; ADG, average daily gain; MBW, mid-test metabolic body weight; YW, yearling weight; KR, Kleiber ratio; RGR, relative growth rate.

**Table 3:** Genetic (±SE, above diagonal) and phenotypic correlations (±SE, below diagonal) among the carcass traits of Hanwoo cattle

Trait	BT	CW	EMA	MS
BT	-	0.18 (0.07)	−0.11 (0.08)	−0.03 (0.08)
CW	0.30 (0.01)	-	0.55 (0.06)	0.16 (0.08)
EMA	0.03 (0.02)	0.57 (0.01)	-	0.28 (0.08)
MS	0.07 (0.02)	0.11 (0.02)	0.21 (0.02)	-

BT, backfat thickness; CW, carcass weight; EMA, eye muscle area; MS, marbling score.

**Table 4:** Genetic (±SE, top) and phenotypic correlations (±SE, bottom) of the carcass traits with growth and related feed efficiency traits in Hanwoo cattle

Trait	BT	CW	EMA	MS
Genetic correlation				
ADG	0.06 (0.08)	0.94 (0.01)	0.58 (0.06)	0.19 (0.08)
MBW	0.11 (0.08)	0.85 (0.02)	0.40 (0.07)	−0.10 (0.08)
YW	0.00 (0.08)	0.74 (0.04)	0.33 (0.07)	−0.16 (0.08)
KR	−0.02 (0.09)	0.47 (0.07)	0.44 (0.08)	0.37 (0.09)
RGR	−0.11 (0.09)	0.14 (0.09)	0.27 (0.09)	0.37 (0.09)
Phenotypic correlation				
ADG	0.17 (0.01)	0.80 (0.01)	0.47 (0.01)	0.04 (0.02)
MBW	0.24 (0.02)	0.84 (0.00)	0.46 (0.01)	0.01 (0.02)
YW	0.19 (0.01)	0.70 (0.01)	0.37 (0.01)	0.01 (0.02)
KR	0.01 (0.02)	0.26 (0.01)	0.18 (0.02)	0.05 (0.02)
RGR	−0.08 (0.02)	−0.03 (0.01)	0.02 (0.02)	0.04 (0.02)

SE, standard error; BT, backfat thickness; CW, carcass weight; EMA, eye muscle area; MS, marbling score; ADG, average daily gain; MBW, mid-test metabolic body weight; YW, yearling weight; KR, Kleiber ratio; RGR, relative growth rate.

**Table 5:** Genetic (±SE, above diagonal) and phenotypic correlations (±SE, below diagonal) between the related feed efficiency and growth traits of Hanwoo cattle

Trait	ADG	MBW	YW	KR	RGR
ADG	-	0.70 (0.05)	0.61 (0.06)	0.70 (0.05)	0.40 (0.08)
MBW	0.63 (0.01)	-	0.89 (0.02)	−0.02 (0.09)	−0.36 (0.08)
YW	0.49 (0.01)	0.85 (0.00)	-	−0.05 (0.09)	−0.30 (0.08)
KR	0.71 (0.01)	−0.09 (0.01)	−0.14 (0.01)	-	0.92 (0.02)
RGR	0.45 (0.01)	−0.37 (0.01)	−0.30 (0.01)	0.92 (0.00)	-

SE, standard error; ADG, average daily gain; MBW, mid-test metabolic body weight; YW, yearling weight; KR, Kleiber ratio; RGR, relative growth rate.
