# Supplementary material for: Genetic parameters and correlations of related feed efficiency, growth, and carcass traits in Hanwoo beef cattle
Source: Anim Biosci. 2020 Aug 30;34(5):824–32. doi: 10.5713/ajas.20.0135 (PMC8100466; doi:10.5713/ajas.20.0135)
Supplement: Supplementary file 1 [file ajas-20-0135-suppl.pdf]

Table S1. Additive genetic variances (σ^2_a), environmental variances (σ^2_e), phenotypic variances (σ^2_p), heritability (h^2), and coefficients of genetic variation (CV_g) estimates (±SEs) for carcass, growth, and related feed efficiency traits using single-trait animal model in Hanwoo cattle

**Trait[1]**	$\boldsymbol{\sigma^2_a}$	$\boldsymbol{\sigma^2_e}$	$\boldsymbol{\sigma^2_p}$	$\boldsymbol{h^2}$	$\boldsymbol{CV_g}$ **(%)**
BT	5.43 (0.59)	5.62 (0.47)	11.06 (0.25)	0.49 (0.05)	26.75
CW	313.17 (45.24)	711.91 (39.12)	1025.10 (21.15)	0.31 (0.04)	5.14
EMA	27.04 (3.21)	35.34 (2.60)	62.38 (1.39)	0.43 (0.05)	6.59
MS	1.50 (0.17)	0.97 (0.13)	2.47 (0.07)	0.61 (0.06)	36.78
ADG	2462.80 (338.34)	5880.20 (293.91)	8343.00 (164.29)	0.30 (0.04)	5.86
MBW	11.85 (1.74)	31.64 (1.53)	43.49 (0.84)	0.27 (0.04)	3.97
YW	267.37 (25.31)	765.09 (20.44)	1032.50 (13.76)	0.26 (0.02)	4.78
KR	0.21 (0.03)	0.49 (0.03)	0.69 (0.01)	0.30 (0.04)	4.69
RGR	6.76 (0.95)	14.69 (0.81)	21.44 (0.43)	0.31 (0.04)	6.47

[1] BT: backfat thickness, CW: carcass weight, EMA: eye muscle area, MS: marbling score, ADG: average daily gain, MBW: mid-test metabolic body weight, YW: yearling weight, KR: Kleiber ratio, RGR: relative growth rate.
